# Factors associated with the impact of quality improvement collaboratives in mental healthcare: An exploratory study

**DOI:** 10.1186/1748-5908-7-1

**Published:** 2012-01-09

**Authors:** Marleen H Versteeg, Miranda GH Laurant, Gerdien C Franx, Annelies J Jacobs, Michel JP Wensing

**Affiliations:** 1 Scientific Institute for Quality of Healthcare (IQ healthcare), Radboud University Nijmegen Medical Centre, P.O. Box 9101, 114 IQ healthcare, 6500 HB Nijmegen, The Netherlands; 2 Trimbos Institute, Da Costakade 45, 3521 VS Utrecht, Postbus 725, 3500 AS Utrecht, The Netherlands

## Abstract

**Background:**

Quality improvement collaboratives (QICs) bring together groups of healthcare professionals to work in a structured manner to improve the quality of healthcare delivery within particular domains. We explored which characteristics of the composition, participation, functioning, and organization of these collaboratives related to changes in the healthcare for patients with anxiety disorders, dual diagnosis, or schizophrenia.

**Methods:**

We studied three QICs involving 29 quality improvement (QI) teams representing a number of mental healthcare organizations in the Netherlands. The aims of the three QICs were the implementation of multidisciplinary practice guidelines in the domains of anxiety disorders, dual diagnosis, and schizophrenia, respectively. We used eight performance indicators to assess the impact of the QI teams on self-reported patient outcomes and process of care outcomes for 1,346 patients. The QI team members completed a questionnaire on the characteristics of the composition, participation in a national program, functioning, and organizational context for their teams. It was expected that an association would be found between these team characteristics and the quality of care for patients with anxiety disorders, dual diagnosis, and schizophrenia.

**Results:**

No consistent patterns of association emerged. Theory-based factors did not perform better than practice-based factors. However, QI teams that received support from their management and both active and inspirational team leadership showed better results. Rather surprisingly, a lower average level of education among the team members was associated with better results, although less consistently than the management and leadership characteristics. Team views with regard to the QI goals of the team and attitudes towards multidisciplinary practice guidelines did not correlate with team success.

**Conclusions:**

No general conclusions about the impact of the characteristics of QI teams on the quality of healthcare can be drawn, but support of the management and active, inspirational team leadership appear to be important. Not only patient outcomes but also the performance indicators of monitoring and screening/assessment showed improvement in many but not all of the QI teams with such characteristics. More studies are needed to identify factors associated with the impact of multidisciplinary practice guidelines in mental healthcare.

## Background

Healthcare providers worldwide are looking to improve the quality of healthcare delivery. Quality improvement collaboratives (QICs) are currently being established on a widespread basis for this purpose. QICs bring together groups of healthcare providers to work in a structured manner to improve the quality of healthcare delivery in a specific domain. Ideal domains for QICs involve interventions derived from evidence-based guidelines or, when these are not available, other best-practice information [1].

There is considerable variation in how QICs are structured and run [2]. A well-known approach consists of the adoption of the Breakthrough Series (BTS) developed and promoted by the Institute for Healthcare Improvement in the United States [3,4]. Although QICs vary in their approach, generally five essential features are involved. There is: a specific topic; clinical and quality improvement experts provide ideas and support for improvement; multi-professional teams from multiple organizations participate; a specific model is used for improvement (*e.g*., set targets, collect data, test for change) [5]; and a series of structured collaborative activities is undertaken (*e.g*., national conferences) [6].

The establishment of QICs is a popular strategy with positive but limited evidence regarding its effectiveness [6,7]. The domain of the QIC, the methods used, the organizational context, and the general healthcare setting can all influence the effectiveness of a particular QIC [1,2,8,9]. In addition, the effects of QICs in mental healthcare settings are largely unknown. Insight into the QI process in the field of mental health care is therefore needed to identify which factors promote the success of QICs and thereby improved mental healthcare [6,7].

In the present study, we evaluated the impact of QICs that are part of a nationwide program to improve mental health care in the Netherlands. The domains of the QICs were the implementation of evidence-based multidisciplinary practice guidelines for anxiety disorders, dual diagnosis, and schizophrenia [10-12]. We also examined if such factors as the composition, participation, functioning, and organizational context for QI teams related to reported quality of healthcare for patients with anxiety disorders, dual diagnosis, or schizophrenia [1,2,9,13-26].

## Methods

### Setting and study population

In 2004, QICs were introduced into mental healthcare in the Netherlands. The domains of depression and schizophrenia were targeted [27]. Given positive evaluations, a nationwide QI program was introduced in the field of mental healthcare in the Netherlands between 2006 and 2009. At the same time a study was designed to evaluate the outcomes of the program.

In this one-year study, three QICs involving a total of 29 QI teams from different mental healthcare organizations—including eight primary healthcare organizations—in the Netherlands were evaluated. Participation in the QICs was voluntary. The participating organizations paid a fee for external support.

The QI teams involved 5 to 13 people, including the team leader. They targeted improved implementation of one of the three multidisciplinary practice guidelines. The QI teams consisted of psychiatrists or general practitioners (only in the domain of anxiety disorders), psychologists or psychotherapists, social workers, physiotherapists, social psychiatric nurses or mental health nurses, and practice assistants.

### Aims of the QICs and national QI program

In promoting the implementation of multidisciplinary practice guidelines, the QICs specifically aimed to improve the delivery of care, monitoring of clinical status, and—in the end—health outcomes. The interventions and instruments used to do this were derived from the evidence reported in the multidisciplinary practice guidelines.

As part of the national QI program, each QIC had one multidisciplinary practice guideline as its topic. This meant that the QI teams in the QIC for anxiety disorders, for example, implemented a stepped care model to achieve better screening and treatment for patients with anxiety disorders. A BTS approach was adopted to do this.

The general goal of the national QI program was to stimulate organizations in mental healthcare to work on quality improvement. Clinical experts therefore defined performance indicators for patient outcomes and process of care outcomes. These indicators were used to assess the impact of the efforts of the QICs and QI teams. The indicators also represented the standard QI teams had to meet to be considered successful at the end of the study (Table 1).

**Table 1:** Performance indicators as predefined by clinical experts with the standard QI teams had to meet at the end of the study

**Patient Outcomes::**

After six months of treatment, 50% of the patients with severe anxiety disorders have a score of less than three on the Clinical Global Impression- Severity (CGI-S) scale.

Experienced quality of life, as measured by the Manchester Short Assessment of Quality of Life (MANSA) scale, improved by 15 points for 75% of patients with a dual diagnosis.

Social functioning, as measured by the Health of the Nation Outcome Scale (HONOS), improved by 2 points for 100% of patients with schizophrenia.

**Monitoring:**

At least 80% of the patients with anxiety disorders are monitored (every six weeks from the start of study and at least two times) using the Clinical Global Impression- Severity (CGI-S) scale.

The quality of life of 100% of the patients with a dual diagnosis is monitored (every 12 weeks and at least two times during the study) using the Manchester Short Assessment of Quality of Life (MANSA) scale.

The social functioning of 100% of the patients with schizophrenia is monitored at least two times using the Health of the Nation Outcome Scale (HONOS).

**Screening/assessment:**

Every month at least 4 out of 1000 patients suspected of anxiety disorders, are screened using the Four Dimensional Symptom Questionnaire (4DSQ).

Of all patients with dual diagnosis, 100% are screened for psychopathology with the Health of the Nation Outcome Scale (HONOS).

During a period of one year, clinical experts and quality improvement experts provided QI support via team visits, conferences, and an internet forum. At least four conferences were organized to provide explicit instruction on techniques for QI and the measurement of QI. The conferences also gave the QI teams an opportunity to exchange knowledge and experiences.

### Outcome measures

Each QI team collected information on patient outcomes (see Data Analysis) and such process of care outcomes as monitoring and screening/assessment (see Data Analysis) in order to determine the impact of their guideline implementation. The QI teams used standardized spreadsheets to register individual patient data across a one-year period. The number of measuring occasions needed to calculate a performance indicator varied. For example, the performance indicator for screening/assessment of patients with dual diagnosis could be calculated on the basis of a single measurement occasion per patient. In contrast, patient outcomes and monitoring rates required at least two measurement occasions per patient. All of the QI teams worked to improve patient outcomes and monitoring rates. The teams in the domains of anxiety disorders and dual diagnosis also worked to improve screening/assessment rates (Table 1).

In addition, questionnaires with self-designed and previously validated items [18,20,23,24] were administered to all members of the QI teams in order to identify factors influencing the efficacy of the QI teams and quality of mental healthcare. Specific hypotheses were formulated with regard to the roles of: team composition, participation in the national program, functioning, and organizational context (Table 2).

**Table 2:** Factors hypothesized (*i.e*., average per QI team) to be associated with successful team performance

**QI teams will perform better if they:**

*Composition characteristics*

have a higher age, more members [2] and more varied types of professionals [13].

are more professional; have a higher level of education, more specialized training, more years of practice on the current job and more years of working in the present organization [14].

spend more time on the improvement [15].

have prior experience with quality improvement [2].

*Participation characteristics*

have a higher attendance of recommended national conferences [16].

*Functioning characteristics*

have more positive attitudes towards the method for improvement (BTS approach), more positive opinions about how their contribution to the improvement is valued by other team members, and more positive opinions about the team's improvement efficacy [17].

have a more positive attitude towards change [18].

have a positive team climate; a positive view about communication with regard to the innovation; agree on the goals of the QIC; and have a positive opinion with regard to the openness of the working method used [19-22].

have a more positive attitude towards evidence-based practice guidelines [23].

*Organizational context characteristics*

receive more organizational support: time, workforce, sponsoring and skills [1,2,9].

receive support from the management of the organization [25].

have an active, inspirational team leader [24,26].

### Composition of the QI team

We collected data on personal characteristics like age [2] and profession. The QI team members were asked about their professional experience in terms of level of education [13,14] and specialized knowledge [14]. These were measured using a nominal scale. Education was divided into: associate degree (general psychiatric nurses, practice assistants), bachelor degree (specialized psychiatric nurses, social workers, physiotherapists), or master's degree (psychiatrists, general practitioners, psychologists, psychotherapists). We also collected data on years of experience with current job and years of employment in present organization [14]. These were continuous variables. The QI team members were further asked about the time spent on improvement (continuous variable) [15]. We measured prior experience with quality improvement [2] by asking QI team members dichotomous questions (yes/no) about: their involvement in other quality improvement activities; their experience with the organization of refresher courses; the development of guidelines; and in the organization of activities for their professional group.

### Participation of the QI team in the national program

To measure participation of the QI teams in the national conferences, they were asked to indicate how many conferences they had attended [16]. Conference attendance by team leaders versus team members was distinguished because the leaders were expected to attend more conferences.

### Functioning of the QI team

The Attitude Social influence Efficacy model (ASE model) [17] was used to examine the perceptions and opinions of the QI team members with regard to their social influence, efficacy and the BTS approach. The Evidence-based Practice Attitude Scale (EBPA) [18] was used to assess the attitudes of the QI team members towards changes in their professional routines. The answers on a five-point Likert scale could range from strongly disagree (= 1) to strongly agree (= 5).

To assess team climate (TCI) [19-22], the QI team members were asked about: communication and readiness for innovation within the team; their views on the goals of the QI team; and their use of the team's approach and/or working method. Response options varied from strongly disagree (= 1) to strongly agree (= 5) for the team's communication and goals; from sometimes (= 1) to often (= 5) for the team's approach or working methods.

Finally, the QI team members were asked about their attitudes towards the evidence-based practice guideline targeted by them, in particular the openness of the guideline to interpretation [23]. The responses on this five-point Likert scale could range from strongly disagree (= 1) to strongly agree (= 5).

### Organizational context of the QI team

Perceptions of the organizational conditions for the QI teams were probed and, in particular, perceptions of the presence of organizational conditions needed for healthcare improvement efforts like time, workforce, sponsoring, and skills [1,2,9]. In addition, we asked if the QI team members experienced support from their managements [25]. A seven-point Likert scale was used, which meant that responding could range from strongly disagree (= 1) to strongly agree (= 7).

Finally, we asked the QI team members about the type of leadership they received from the QI team leader. Did they perceive the leader as inspirational? transformational? passive? The responses on the Multi Factorial Leadership Questionnaire (MFLQ) [24,26] could vary from sometimes (= 1) to often (= 5).

### Data analysis

For the QIC in the domain of anxiety disorders and the QIC in the domain of dual diagnosis, three performance indicators were selected to assess the impact of improvement efforts: one for patient outcomes, one for monitoring, and one for screening/assessment. For the QIC in the domain of schizophrenia, two performance indicators were selected: one for patient outcomes and one for monitoring. Only QI teams with at least two months of data registration were included in the analysis. If a second measurement occasion was needed for the indicator but was missing for a patient, the patient was excluded from the analysis. The mean scores for the indicators were calculated for each QI team.

After a period of one year, only a few of the QI teams met the standards that the clinical experts had pre-defined. We therefore decided to measure improvement relative to baseline and assumed the baseline scores for the performance indicators to be zero, as the multidisciplinary guidelines had not yet been implemented at baseline. Patient outcomes and process of care outcomes were measured using the Clinical Global Impression-Severity (CGI-S), Manchester Short Assessment of Quality of Life (MANSA), Health of the Nation Outcome Scale (HONOS), and the Four Dimensional Symptom Questionnaire (4DSQ) (Table 1). We then identified those QI teams with the most and least improvement for the different performance indicators within each QIC: Those teams with change scores in the lowest quartile (≤ 25%) were considered least successful; those teams with change scores in the highest quartile (≥ 75%) were considered most successful. Depending on the number of QI teams participating in the QIC, two or three teams could be identified as least and most successful, respectively. Next we calculated the mean impact of the most and least successful QI teams.

Finally we calculated the mean scores of the characteristics (related to the composition, participation, functioning, and organizational context) for the QI teams in the lowest and highest quartiles.

Because our numbers were too low for statistical testing and the sampling was not random, we decided to consider a factor as possibly associated with the impact of a team when the following three conditions were met: the mean scores for the factor from the most successful QI teams did not fall within the range of the scores from the least successful QI teams; the mean scores for the factors from the least successful QI teams did not fall within the range of the scores from the most successful QI teams; and both these conditions were met for at least two of the performance indicators or in at least two of the three QICs.

We then examined how the QI team factors of composition, participation, functioning, and organizational context related to the performance indicators of patient outcome, monitoring, and screening/assessment to identify possibly positive, negative, or possibly mixed associations. We examined major associations between QI team factors and performance indicators (Table 3) and associations between characteristics of QI teams and performance indicators (Table 4).

**Table 3:** Major associations between QI team factors (*i.e*., composition, participation, functioning and the 0rganizational context) and performance indicators (*i.e*., patient outcomes, monitoring, screening/assessment) for successful teams

	Anxiety disorders	Dual diagnosis	Schizo phrenia	*Anxiety disorders*	*Dual diagnosis*	*Schizo* *phrenia*	Anxiety disorders	Dual diagnosis
	
	Patient outcomes			*Monitoring*			Screening/assessment	
Team composition	**+/-**	**+/-**	**+/-**	**+/-**	**+/-**	**+/-**	**+/-**	**+/-**

Participation in the national program		**+**				**+**	**-**	

Team Functioning	**-**	**+**	**-**		**+**	**+/-**	**-**	**+/-**

Organizational context	**+/-**	**+/-**	**+**	**+/-**	**+**	**+**	**+/-**	

**+ **= Positive association, **- **= negative association, **+/- **mixed association, empty cell = no association

**Table 4:** Associations between characteristics of QI teams (*i.e*., composition, participation, functioning and the organizational context) and performance indicators (*i.e*., patient outcomes, monitoring, screening/assessment) for successful teams

	Anxiety disorders	Dual diagnosis	Schizo phrenia	*Anxiety disorders*	*Dual* *diagnosis*	*Schizo* *phrenia*	Anxiety disorders	Dual diagnosis
	
	Patient outcomes			*Monitoring*			Screening/assessment	
*Team Composition*								

Average age	**+**						**-**	**-**

Number of team members	**-**				**+**	**+**		

Number of different professionals	**+**							**-**

Level of education:								

% Master degree		**-**			**-**	**-**		**-**

% Bachelor degree					**-**			**+**

% Associate degree		**+**			**+**			

Years of practice in this job	**+**		**-**	**+**	**+**		**-**	

Years of practice in this organization							**-**	**-**

Number of team members with specialized knowledge	**+**					**+**	**-**	

Time spent on improvement	**-**		**+**					

% Involvement in quality improvement			**+**	**-**		**-**	**+**	**-**

*Participation in the national program*								

% Attendance conferences QI team members		**+**			**+**			

% Attendance conferences by QI team leaders		**+**			**+**	**-**		

*Team functioning*								

Social influence		**+**				**-**		

Efficacy	**-**		**-**					

Attitude			**-**					**-**

Attitude quality improvement		**+**			**+**		**-**	

Communication/innovation		**+**				**+**		

Targets								

Approach-working method		**+**	**-**				**-**	

Attitude guidelines, factor innovation (specificity/flexibility)								**+**

*Organizational context*								

Organizational conditions present		**+**			**+**			

Support management		**+**		**+**	**+**		**+**	

Type of leadership:								

Inspirational leadership	**+**	**+**		**+**		**+**		

Transactional leadership						**+**		

Passive leadership	**-**	**-**	**+**	**-**			**-**	

**+ **= Positive association, **- **= negative association, empty cell = no association

## Results

### Final study population

In the end, 26 QI teams of 19 mental healthcare organizations were included in the analysis. Three QI teams were excluded because they did not register patient data for the required two months. One organization participated in two QICs. Three organizations participated with more than one QI team in a QIC. We included: 12 QI teams in the QIC on anxiety disorders; eight in the QIC for schizophrenia; and six in the QIC for dual diagnoses. Most of the QI teams represented clinical mental healthcare settings; eight of 12 teams for anxiety disorders represented primary care settings (Table 5).

**Table 5:** Characteristics of the QI teams per QIC (N = 26)

	Anxiety disorders	Schizo- phrenia	Dual diagnosis	Total
Number of QI teams	12	8	6	26

Number of organizations represented by QI teams	7	7	5	19

Number of questionnaires sent	82	72	75	229

Respondents/known non- respondents^#^	63/11	47/13	43/17	153/41

Response	76.8%	65.3%	57.3%	66.8%

Mean number of members per QI team (range amongst QI teams)	6.2 (5 to 8)	7.5 (3 to 13)	10 (7 to 13)	7.5 (3 to 13)

Percentage male	43.2%	48.3%	33.3%	41.8%

Mean age per QI team (range amongst QI teams)	44.2 (25 to 62)	42.8 (25 to 59)	38.4 (22 to 57)	42.0 (22 to 62)

Number of patients reached (range amongst QI teams)	571 (10 to 165)	467 (38 to 82)	308 (11 to 87)	1346 (1 to 165)

^#^) Of 41 non-respondents age and sex were known, this was included in this table

### Impact of the QI teams on healthcare improvement

The actual measurement period for the teams varied from 2 to 12 months. In the QIC for anxiety disorders, the period ranged from 7 to 12 months; in the QIC for schizophrenia, six teams of the eight teams registered patient data for more than eight months; and in the QIC for dual diagnosis, the period ranged from 2 to 8 months. As the measurement period proceeded, the number of registrations per patient declined in all of the QI teams.

In the end, four QI teams were successful on at least two of the performance indicators: For dual diagnosis one QI team was successful on all three performance indicators; two QI teams for anxiety disorders and another dual diagnosis QI team were successful on patient and monitoring outcomes.

In Table 6 and below, we summarize the impact of the QI teams from the different QICs (*i.e*., domains for improvement) on the performance indicators (*i.e*., patient outcome, monitoring, and screening/assessment).

**Table 6:** Impact of the QI teams on performance indicators in the QIC domains of anxiety disorders, dual diagnosis, and schizophrenia

	Performance indicators:					
	
	Patient outcomes		Monitoring		Screening/assessment	
	
	Mean	Range amongst QI teams	Mean	Range amongst QI teams	Mean	Range amongst QI teams
Anxiety disorders (N = 12)	8.0%	0 to 23.9%	35.9%	7.9% to 78.6%	1.4‰	0.9‰ to 2.3^-^‰

Dual diagnosis (N = 6)	8.6%	0 to 27.3%	5.8%	0 to 28.9%	33.7%	9.1 to 82.8%

Schizophrenia (N = 8)	13.3%	2.1% to 23.3%	48.8%	52.6% to 98.0%	n.a.^#^	n.a.^#^

^#^) Not applicable

### Patient outcomes

After six months of treatment, 8% of the patients with severe anxiety disorders were less anxious (range 0 to 23.9% amongst QI teams). The social functioning of the patients with schizophrenia improved with 13.3% (range 2.1% to 23.3%). And 8.6% of the patients with a dual diagnosis experienced an improved quality of life (range 0 to 27.3%).

### Process of care outcomes: monitoring

The QI teams for anxiety disorders monitored the severity of condition for 35.9% of their patients (range 7.9% to 78.6% amongst QI teams). The social functioning of 48.8% of the patients suffering from schizophrenia was systematically monitored (range 52.6 to 98.0%). The QI teams for dual diagnosis monitored the quality of life for 5.8% of their patients (range 0 to 28.9%).

### Process of care outcomes: screening/assessment

Eight QI teams of anxiety disorders executed goals on screening and assessment with the 4DSQ. These primary care teams screened 1.4‰ of patients per practice (range 0.9‰ to2.3‰ amongst QI teams). The QI teams for dual diagnosis screened 33.7% of patients for psychopathology using the HONOS (range 9.1% to 82.8%). The QIC for schizophrenia had no screening/assessment goals.

### Questionnaire response rate

A total of 229 QI team members/leaders were sent a questionnaire to gain insight into the characteristics of the QI teams. The response rate was 66.8%. We also gathered data on the demographic characteristics of the 41 QI team leaders who did not respond (*i.e*., return the questionnaire) (Table 5). For previously validated questionnaire items [18,20,23,24] we calculated the Cronbach's α (Table 7).

**Table 7:** Cronbach's α of the validated questionnaire items measuring characteristics of the QI teams

		Anxiety disorders	Dual Diagnosis	Schizo phrenia
ASE model	Social influence	0.852	0.847	0.749
	
	Efficacy	0.513	0.470	0.496
	
	Attitude	0.661	0.617	0.691

Attitude quality improvement (EBPA)	Openness (= attitude towards change in working routine)	0.745	0.942	0.770

Team Climate Inventory (TCI)	Communication/ Innovation	0.778	0.773	0.764
	
	Targets	0.754	0.755	0.759
	
	Approach	0.877	0.873	0.635

Organizational Conditions	Time/workforce/ sponsoring/skills	0.825	0.781	0.806

Type of leadership (MFLQ)	Inspirational leadership	0.951	0.958	0.964
	
	Transactional leadership	0.709	0.866	0.780
	
	Passive leadership	0.733	0.629	0.735

Attitude regarding guideline	Factor innovation (specificity/flexibility)	0.591	0.887	0.429

### Associations between QI team factors and performance indicators

In Table 3, the major associations between QI team factors (*i.e*., composition, participation in national program, functioning, and organizational context) and the performance indicators we measured (*i.e*., patient outcome, monitoring, screening/assessment) are presented. The direction of the influence of the factors is noted. The results are summarized below.

### Team composition

All the possible associations in the composition of the successful QI teams were either mixed or absent.

### Participation the national program

Only the participation of the most successful QI teams from the dual diagnosis QIC showed a possible positive association with patient outcomes and monitoring. The participation of the most successful QI teams from the schizophrenia QIC appeared to negatively affect monitoring.

### Team functioning

The team functioning of the most successful QI teams appeared to be associated with the following outcomes: the functioning of the QI teams within the dual diagnosis QIC appeared to promote patient outcomes and monitoring; for anxiety disorders, team functioning appeared to negatively affect patient outcomes and screening/assessment; and the functioning of the QI teams within the schizophrenia QIC also appeared to negatively affect patient outcomes. Other associations were either mixed or absent.

### Organizational context

The organizational context for the most successful QI teams appeared to be associated with better patient outcomes and greater monitoring in the domain of schizophrenia and greater monitoring in the domain of dual diagnosis. Other associations were either mixed or absent.

### Associations between characteristics of the QI teams and performance indicators

In Table 4, we report the possible associations between 22 characteristics of the QI teams and the performance indicators: patient outcome, monitoring, and screening/assessment. The direction of the associations is also indicated, and the results are discussed further below. The findings for the least and most successful QI teams in the domains of dual diagnosis, depression, and schizophrenia are available in additional files (Additional Files 1, 2, and 3).

Out of nine characteristics of QI team composition, two appeared to relate to team success: years of employment in the organization and the level of education.

QI teams that had worked fewer years in the organization they represented showed better results for screening/assessment in the domains of anxiety disorders (average 5.3 years, range 4.7 to 5.8 years amongst QI teams) and dual diagnosis (average 3.9 years, range 3.9 to 3.9 years).

QI teams that counted more members with an associate degree and fewer members with a master's degree had better results. They showed better patient outcomes, greater monitoring, and screening/assessment within the domain of dual diagnosis. The successful teams consisted of fewer psychiatrists, general practitioners, psychologists, and psychotherapists (average 12.3%, range 9.1 to 15.4%, average 22% amongst QI teams, range 15.4 to 28.6% amongst QI teams, respectively). A similar pattern was found for the most successful QI teams within the domain of schizophrenia (average 16.1%, range 15.4 to 16.7%). They showed greater monitoring.

The successful QI teams in the domain of dual diagnosis also consisted of more mental health nurses and practice assistants (average 34.3%, range 23.1 to 45.5%) they showed better patient outcomes and greater monitoring.

### Characteristics of team participation in national program

A higher average attendance of the recommended conferences appeared to be associated with better results in the domain of dual diagnosis in particular (average attendance 77.5%, range 67.5 to 87.5% amongst QI teams). The successful QI teams showed better patient outcomes and greater monitoring.

### Characteristics of team functioning

Out of eight characteristics of QI team functioning, three appeared to be associated with improvement: views about the team's QI efficacy; the attitudes towards change; and views about communication with regard to the innovation within the QI team.

Those QI teams with less positive perceptions about their QI efficacy showed better performance results. Better patient outcomes were found for the QI teams with less positive efficacy expectations in the domains of anxiety disorders (average item score 3.2, range 3.2 to 3.3 amongst QI teams) and schizophrenia (average item score 3.1, range 3.0 to 3.2).

Those QI teams with less positive attitudes towards change also showed better results in some domains. In the domain of schizophrenia, those QI teams with less positive attitudes towards change (average item score 3.5, range 3.5 to 3.5 amongst QI teams) showed better patient outcomes. In the domain of dual diagnosis, the QI teams with less positive attitudes towards change (average item score 3.7, range 3.4 to 3.9) showed greater screening/assessment. Finally, the QI teams with positive views about communication with regard to the innovation in the domain of dual diagnosis (average item score3.5, range 3.4 to 3.6) showed better patient outcomes; those in the domain of schizophrenia (average item score 4.1, range 4.0 to 4.1) showed greater monitoring.

### Characteristics of the organizational context

All characteristics—the presence of organizational conditions for improvement, support from the management, and type of leadership—appeared to be associated with better patient outcomes and process of care outcomes.

QI teams with enough time, workforce, sponsoring, and skills generally showed better results. The QI teams with good organizational conditions in the domain of dual diagnosis (average item score 4.6, range 4.4 to 4.8 amongst QI teams) showed better patient outcomes and greater monitoring.

Similarly, those that felt supported by their management in the domain of dual diagnosis (average item score 5.6, range 5.3 to 5.8 amongst QI teams) showed relatively better patient outcomes and greater monitoring. Also in the domain of anxiety disorders, the successful QI teams that felt supported by the management of the organization (average item score 4.4, range 4.0 to 4.9, average item score 4.4, range 4.2 to 4.5, respectively) showed greater monitoring and screening/assessment.

QI teams with an inspiring leader showed better results [24]. The successful teams with particularly inspirational leadership in the domain of anxiety disorders (average item score 3.6, range 3.4 to 3.9, average item score 3.9, range 3.4 to 4.3 amongst QI teams, respectively) showed better patient outcomes and greater monitoring. The successful teams in the domain of schizophrenia (average item score 4.2, range 3.8 to 4.5) showed greater monitoring. And the successful QI teams in the domain of dual diagnosis (average item score 4.0, range 3.9 to 4.1) showed better patient outcomes.

Finally, those QI teams with a more active leader (*i.e*., a lower average item score) showed better results as well. The successful QI teams with active leadership in the domain of anxiety disorders (average item score 1.8, range 1.0 to 2.2 and an average item score 1.8, range 1.5 to 2.0 amongst QI teams, respectively) showed improvement on all performance indicators (*i.e*., patient outcomes, monitoring, and screening/assessment). The successful QI teams with active leadership in the domain of dual diagnosis (average item score 1.8, range 1.7 to 1.8) showed relatively better patient outcomes.

The successful QI teams in the domain of schizophrenia had an average item score of 2.8 (range 2.5 to 3.1), which matched the minimum score needed to be considered a team with an active leader as proposed by Den Hartog [24]; they showed better patient outcomes.

## Discussion

In this study, we explored which aspects of the composition, participation, functioning, and organizational contexts for QI teams appeared to be associated with improved quality of mental healthcare. The impact of QI teams improvements efforts to promote the implementation of multidisciplinary evidence-based guidelines and recommendations were examined in the domains of anxiety disorders, dual diagnosis, and schizophrenia.

A general clear pattern of results could not be detected. Factors derived from theory did not perform better than factors derived from practice. Only modest support was found for a few select factors but not on a widespread basis. Support from the management of the organization and active, inspirational QI team leadership related to somewhat more than 50% or the performance outcomes for the most successful QI teams in the domains we studied. In other research evaluating a national program aimed at quality improvement in Dutch hospitals [28], support from the management was also found to be an important factor for success.

Less consistently, factors like average level of education in the QI team, number of years at current job, and previous involvement in QI efforts appeared to be associated with improved quality of mental healthcare.

QI teams with more members with an associate degree and fewer members with a master's degree surprisingly had better results. It is possible that QI teams need members from a variety of backgrounds and thus not only members with strong theoretical backgrounds (*i.e*., members with a master's degree) [14] to be successful. QI teams need professionals who are able to take control of the improvement initiative, supervise the improvement process, and record data or, in other words, members with an associate degree who tend to be general nurses [6].

The number of years of practice (*i.e*., on the current job and prior involvement in QI efforts showed possible associations with at least 50% of the performance indicators for the successful QI teams. Although the directions of the associations were mixed, purely the number of associations suggests that these factors play a role in the efforts of teams to improve the quality of mental healthcare.

The present findings are in keeping with the findings summarized in reviews by Hulscher and Schouten [6,7] who reported only a few factors to appear to influence the effectiveness of QICs. Comparison of QI teams within specific QICs (limited to one domain) is therefore called for, using a mixed approach that thus combines both quantitative and qualitative methods to better understand improvement processes in particularly the field of mental healthcare [6,29].

### Strengths and weaknesses of the study

A strong point of the present study is that it was conducted close to daily mental healthcare practice. The findings are thus likely to reflect real people during real practice.

Most of the evaluations of QICs to date have been based on general hospitals and nursing homes, which makes the present findings a welcome contribution. That is, the present results contribute to our knowledge of the impact of QICs in general and within the field of mental healthcare in particular.

Limitations on the present study should be mentioned as well. The number of participating QI teams was small and only QI teams with a minimum of two months of data registration were included in the analysis.

Moreover, the amount of registration (*i.e*., data) per patient declined as the study proceeded. No relation, however, was found between QI team success and the amount of data available per team. How we handled missing data (if a second measurement occasion was needed to calculate the performance indicator but was missing for a patient, the patient was excluded from the analysis) produced a conservative estimate of team success, which may mean that the true impact of the QI teams has been underestimated. Also, the range in the number of patients reached and the different number of registered measurements per patient may have affected the decision about the most and least successful QI teams.

With the exception of the screening/assessment rates for patients with anxiety disorders, it is not known whether the denominator for the performance indicator scores corresponds to the number of patients who could be expected in the participating mental healthcare organizations and general practices. The absence of baseline measurements and control groups is yet another limitation on the present study. For some of the improvement measures, the internal consistency—as expressed by the Cronbach's alpha—was only moderate.

In the present research, we opted to study a wide range of factors in conjunction with the efforts of QICs to improve the quality of mental healthcare. To discern a relationship between these factors and improved mental healthcare outcomes, we used a qualitative cross-sectional study design. Because the numbers proved too low for statistical testing and sampling was not random, we were only able to identify possible associations. Further research is therefore needed to confirm the positive findings of our study while addressing limitations.

## Conclusions

The QICs in this study aimed to improve patient care by implementing the recommendations provided by multidisciplinary practice guidelines in three separate domains of mental healthcare. The success of the QICs appears to depend on a range of largely still unknown factors. On the basis of the present research, however, it can be concluded that QI teams should be given active and inspiring team leadership. Support from management of the organization is also important. QI teams should have a variety of members: In addition to professionals with heavy theoretical backgrounds, the teams should include professionals who are willing (and able) to take control of the improvement process, supervise the improvement process, and record the necessary data to do all this. The views of QI team members regarding the goals of the team or the QI effort appear to be of less importance. But the number of years of practice on the current job and prior involvement in QI efforts do appear to be important for successful QI team performance. Additional study is nevertheless needed to identify factors associated with the impact of QICs on the implementation of multidisciplinary practice guidelines. We recommend a mixed approach that thus combines both quantitative and qualitative methods to better understand improvement processes in particularly the field of mental healthcare

## Competing interests

The authors declare that they have no competing interests. Michel Wensing is an Associate Editor of Implementation Science. Another senior Editor made all decisions on this manuscript.

## Authors' contributions

MW wrote the study outline and MV and ML were responsible for designing the study protocol. MV conducted the study, analyzed the data, interpreted the results, drafted the manuscript, critically revised the manuscript, and managed its further revision. ML assisted with the in interpretation of the results and the drafting the manuscript. AJ en MW participated in the design of the study, assisted with the interpretation of the results, helped with critical revision, and helped supervise further revision. GF assisted helped with the interpretation of the results and the critical revision of the manuscript. All authors have read and approved the final manuscript.

## Supplementary Material

Additional file 1 **Table 8 Characteristics of most and least successful QI teams in relation to patient outcomes**. Click here for file

Additional file 2 **Table 9 Characteristics of most and least successful QI teams in relation to monitoring**. Click here for file

Additional file 3 **Table 10 Characteristics of most and least successful QI teams in relation to screening/assessment**. Click here for file
