# Supplementary material for: Factors associated with the impact of quality improvement collaboratives in mental healthcare: An exploratory study
Source: Implement Sci. 2012 Jan 9;7:1. doi: 10.1186/1748-5908-7-1 (PMC3313876; doi:10.1186/1748-5908-7-1)
Supplement: Additional file 1 — Table 8 Characteristics of most and least successful QI teams in relation to patient outcomes. [file 1748-5908-7-1-S1.DOC]

Table 8 Characteristics of most and least successful QI teams in relation to patient outcomes

|  | Anxiety disorders (N=12) | | | | Dual diagnosis (N=6) | | | | Schizophrenia (N=8) | | | |
| --- | --- | --- | --- | --- | --- | --- | --- | --- | --- | --- | --- | --- |
|  | Most successful teams | | Least successful teams | | Most successful  teams | | Least successful teams | | Most successful teams | | Least successful teams | |
|  | Mean | Range | Mean | Range | Mean | Range | Mean | Range | Mean | Range | Mean | Range |
| *Team composition* | | | | | | | | | | | | |
| Average age | 47.3 | 45.0 – 50.8 | 42.5 | 37.5 – 47.0 | 38.3 | 36.6 – 40.0 | 37.9 | 34.5 – 41.3 | 44.4 | 42.8 - 46 | 40 | 35.3 - 45.2 |
| Number of team members | 6.3 | 5.0 – 7.0 | 5.7 | 5.0 – 6.0 | 12.0 | 11.0 – 13.0 | 10.5 | 8.0– 13.0 | 9.5 | 9.0– 10.0 | 8.5 | 4.0 – 13.0 |
| Number of different professionals | 4.7 | 4.0 – 5.0 | 3.7 | 3.0 – 4.0 | 4.0 | 3.0 – 5.0 | 5.0 | 4.0 – 6.0 | 5.0 |  | 4.0 | 2.0 – 6.0 |
| Level of education |  |  |  |  |  |  |  |  |  |  |  |  |
| % Master degree | 44.4 | 33.3-50 | 58.9 | 33.3-83.3 | 12.3 | 9.1-15.4 | 31.0 | 28.6-33.3 | 15.6 | 11.1-20.0 | 24.4 | 15.4 – 33.3 |
| % Bachelor degree | 55.7 | 50.0 – 66.7 | 41.1 | 16.7 – 66.7 | 53.5 | 45.5-61.5 | 52.4 | 33.3-71.4 | 63.9 | 50.0-77.8 | 60.3 | 53.8 – 66.7 |
| % Associate degree | 0 | 0 | 0 | 0 | 34.3 | 23.1-45.5 | 16.7 | 0-33.3 | 15.6 | 11.1-20.0 | 15.4 | 0 - 30.8 |
| Years of practice in this job | 13.6 | 11.6 – 15.4 | 11.4 | 10.6 – 13.1 | 7.3 | 5.7 – 8.8 | 5.1 | 2.5 – 7.7 | 6.4 | 4.8 – 8.0 | 8.3 | 7.0 - 9.5 |
| Years of practice in this organization | 13.9 | 10.6 – 17 | 11.3 | 5.8 – 16 | 7.8 | 3.9 - 11.7 | 6.8 | 3.9 – 9.7 | 10.5 | 4.2 - 16.8 | 11.5 | 9.7 – 13.3 |
| Number of team members with specialized knowledge | 3.3 | 2.0 – 5.0 | 1.7 | 1.0 – 2.0 | 2.5 | 2.0– 3.0 | 2.0 | 1.0 – 3.0 | 3.5 | 2.0 – 5.0 | 2.0 | 0 – 4.0 |
| Time spent on the improvement | 0.11 | 0.09 – 0.14 | 0.27 | 0.02 – 0.7 | 0.12 | 0.1 – 0.14 | 0.10 | 0.07 – 013 | 0.16 | 0.14 - 0.17 | 0.13 | 0.11 - 015 |
| % Involvement in quality improvement | 38.9 | 35.0 – 41.7 | 47.2 | 37.5 – 58.3 | 63.2 | 37.5 - 88.8 | 39.3 | 28.6 – 50.0 | 90.0 | 80.0 - 100 | 47.7 | 28.6 – 66.7 |
| *Participation in national program* | | | | | | | | | | | | |
| % Attendance conferences QI team members | 55.4 | 37.5 – 68.8 | 56.3 | 50.0 – 68.8 | 77.5 | 67.5 – 87.5 | 57.5 | 57.5 | 50.0 | 42.5 - 57.5 | 32.5 | 0 – 65.0 |
| % Attendance of conferences by QI team leaders | 58.3 | 0 – 100 | 50.0 | 0 – 100 | 87.5 | 75.0 – 100 | 37.5 | 0 – 75.0 | 50.0 | 50.0 | 50.0 | 0 - 100 |
| *Team functioning (average item score)* | | | | | | | | | | | | |
| Social influence (ASE) | 4.0 | 3.9 – 4.1 | 4.0 | 3.7 – 4.2 | 3.9 | 3.6 – 4.1 | 3.5 | 3.4 – 3.6 | 4.0 | 3.9 – 4.0 | 4.3 | 4.0 – 4.5 |
| Efficacy (ASE) | 3.2 | 3.2 – 3.3 | 3.7 | 3.6 – 3.7 | 3.2 | 3.0 – 3.4 | 3.4 | 3.3 – 3.4 | 3.1 | 3.0 – 3.2 | 3.5 | 3.4 – 3.6 |
| Attitude (ASE) | 3.6 | 3.5 – 3.8 | 3.7 | 3.5 – 3.9 | 3.8 | 3.6 – 3.9 | 3.6 | 3.4 – 4.1 | 3.5 | 3.5 – 3.5 | 3.9 | 3.8 - 3.9- |
| Attitude quality improvement (EBPA) | 3.9 | 3.9 – 4.0 | 3.8 | 3.5 – 3.9 | 4.2 | 4.1 – 4.3 | 3.9 | 3.8 – 4.0 | 4.1 | 3.9 – 4.3 | 3.9 | 3.7 – 4.0 |
| Communication/ innovation (TCI) | 3.8 | 3.5 – 4 | 3.5 | 2.8 – 4.1 | 3.5 | 3.4 – 3.6 | 3.2 | 3.0 – 3.3 | 3.6 | 3.5 - 3.7 | 3.8 | 3.5 – 4.0 |
| Targets (TCI) | 3.7 | 3.5 – 3.9 | 4.0 | 3.6 – 4.4 | 3.7 | 3.5 – 3.9 | 3.7 | 3.4 – 3.9 | 3.9 | 3.7 – 4.1 | 4.0 | 3.9 - 4.1 |
| Approach- working method (TCI) | 3.8 | 3.6 – 4.2 | 3.5 | 2.9 – 3.9 | 3.3 | 3.2 – 3.3 | 2.8 | 2.6 – 3.0 | 3.3 | 3.2 – 3.3 | 3.8 | 3.6 – 4.0 |
| Attitude guidelines, factor innovation | 7.6 | 0 – 8.0 | 7.4 | 7.0 – 7.8 | 7.8 | 7.5 - 8.0 | 7.9 | 7.8 – 8.0 | 8 .0 | 8.0 | 7.7 | 7.4 – 8.0 |
| *Organizational context(average item score)* | | | | | | | | | | | | |
| Organizational conditions present | 4.3 | 3.8 – 4.6 | 3.6 | 3.6 | 4.6 | 4.4 – 4.8 | 3.6 | 3.1 – 4.1 | 3.8 | 3.1 - 4.5 | 3.9 | 3.5 - 4.2 |
| Support management | 4.0 | 3.0– 4.9 | 2.9 | 1.7 – 4.5 | 5.6 | 5.3 – 5.8 | 4.2 | 3.6 – 4.7 | 4.0 | 3.6 – 4.4 | 2.6 | 4.1 |
| -Inspirational leadership (MFLQ) | 3.6 | 3.4 – 3.9 | 2.9 | 2.0 – 3.5 | 4.0 | 3.9 - 4.1 | 3.3 | 3.2 – 3.4 | 2.4 | 2.3 - 2.4 | 3.8 | 0 - 3.8 |
| -Transactional leadership (MFLQ) | 2.5 | 1.9 – 2.8 | 2.6 | 1.4 – 3.5 | 2.8 | 2.6 – 3.0 | 2.8 | 2.7 – 2.8 | 1.8 | 1.7 – 1.9 | 2.9 | 0 - 2.9 |
| -Passive leadership (MFLQ) | 1.8 | 1.0 – 2.2 | 2.5 | 2.0 – 2.9 | 1.8 | 1.7 – 1.8 | 2.7 | 2.2 – 3.1 | 2.8 | 2.5– 3.1 | 1.8 | 0 - 1.8 |
