# Supplementary material for: Factors associated with the impact of quality improvement collaboratives in mental healthcare: An exploratory study
Source: Implement Sci. 2012 Jan 9;7:1. doi: 10.1186/1748-5908-7-1 (PMC3313876; doi:10.1186/1748-5908-7-1)
Supplement: Additional file 2 — Table 9 Characteristics of most and least successful QI teams in relation to monitoring. [file 1748-5908-7-1-S2.DOC]

Table 9 Characteristics of most and least successful QI teams in relation to monitoring

|  | Anxiety disorders (N=12) | | | | Dual diagnosis (N=6) | | | | Schizophrenia (N=8) | | | |
| --- | --- | --- | --- | --- | --- | --- | --- | --- | --- | --- | --- | --- |
|  | Most successful teams | | Least successful teams | | Most successful  teams | | Least successful teams | | Most successful teams | | Least successful teams | |
|  | Mean | Range | Mean | Range | Mean | Range | Mean | Range | Mean | Range | Mean | Range |
| *Team Composition* | | | | | | | | | | | | |
| Average age | 45.5 | 45.5 | 42.5 | 37.5 – 46.0 | 38.3 | 36.6 – 40 | 38.2 | 34.5 – 41.9 | 41.7 | 38.2 – 45.2 | 39.4 | 35.3 – 43.4 |
| Number of team members | 6.7 | 6.0 – 7.0 | 6.0 | 5.0 – 7.0 | 12.0 | 11.0 – 13.0 | 8.0 | 8.0 | 9.5 | 6.0 - 13.0 | 5.5 | 4.0 – 7.0 |
| Number of different professionals | 4.3 | 4.0 – 5.0 | 4.3 | 4.0 – 5.0 | 4.0 | 3.0 – 5.0 | 4.5 | 4.0 – 5.0 | 4.5 | 3.0 – 6.0 | 3.5 | 2 .0– 5.0 |
| Level of education |  |  |  |  |  |  |  |  |  |  |  |  |
| % Master degree | 52.4 | 50 – 57.1 | 45.4 | 33.3 – 60.0 | 12.3 | 9.1 – 15.4 | 26.8 | 25 – 28.6 | 16.1 | 15.4-16.7 | 31.0 | 28.6 – 33.3 |
| % Bachelor degree | 47.6 | 42.9 – 50 | 54.6 | 40.0 – 66.7 | 53.5 | 45.5 – 61.5 | 67.0 | 62.5 – 71.4 | 68.6 | 53.8 – 83.3 | 61.9 | 57.1- 66.7 |
| % Associate degree | 0 | 0 | 0 | 0 | 34.3 | 23.1 – 45.5 | 6.3 | 0 – 12.5 | 15.4 | 0 – 30.8 | 7.2 | 0 –14.3 |
| Years of practice in this job | 14.4 | 13.8 – 15.4 | 11.4 | 10.6 – 12.1 | 7.3 | 5.7 – 8.8 | 2.3 | 2.1 – 2.5 | 5.8 | 2.0 – 9.5 | 8.0 | 7.0 – 8.9 |
| Years of practice in this organization | 11.3 | 9.4 – 14.0 | 13.9 | 8.6 – 17 | 7.8 | 3.9 – 11.7 | 6.0 | 3.9 -.0 | 9.2 | 5.0 – 13.3 | 11.6 | 9.7 – 13.4 |
| Number of team members with specialized knowledge | 3.7 | 2.0 – 5.0 | 2.7 | 2.0 – 3.0 | 2.5 | 2.0 – 3.0 | 3.0 | 3.0 | 3.5 | 3.0 – 4.0 | 1.5 | 0 – 3.0 |
| Time spent on improvement | 0.09 | 0.06 – 0.11 | 0.1 | 0.02 – 0.14 | 0.12 | 0.1 – 014 | 0.15 | 0.07 – 0.23 | 0.16 | 0.11 – 0.21 | 0.13 | 0.11 – 0.15 |
| % Involvement in quality improvement | 37.5 | 35.0 – 41.7 | 48.2 | 40.0 – 58.3 | 63.2 | 37.5 – 88.8 | 57.2 | 28.6 – 85.7 | 26.8 | 25.0 – 28.6 | 61.9 | 57.1 – 66.7 |
| *Participation in national program* | | | | | | | | | | | | |
| % Attendance conferences QI team members | 60.4 | 37.5 – 75 | 56.7 | 50.0 – 60.0 | 77.5 | 67.5 – 87.5 | 63.8 | 57.5 - 70 | 66.3 | 65.0 - 67.5 | 40.0 | 0 – 80.0 |
| % Attendance of conferences by QI team leaders | 33.3 | 0 – 100 | 66.7 | 50.0 – 7.05 | 87.5 | 75.0 - 100 | 0 | 0 | 25.0 | 0 – 50.0 | 100 | 100 |
| *Team functioning (average item score)* | | | | | | | | | | | | |
| Social influence (ASE) | 4.1 | 4.0 – 4.1 | 3.9 | 3.7 – 4.0 | 3.9 | 3.6 – 4.1 | 3.8 | 3.4 – 4.1 | 3.7 | 3.3 – 4.0 | 4.2 | 3.9 - 4.5 |
| Efficacy (ASE) | 3.4 | 3.2 – 3.6 | 3.5 | 3.2 – 3.7 | 3.2 | 3.0 – 3.4 | 3.2 | 3.1 - 3.3 | 3.4 | 3.3 – 3.4 | 3.4 | 3.1 - 3.6 |
| Attitude (ASE) | 3.6 | 3.5 – 3.8 | 3.5 | 3.5 – 3.6 | 3.8 | 3.6 – 3.9 | 3.7 | 3.4 – 4.0 | 3.6 | 3.3 – 3.9 | 3.7 | 3.4 – 4.0 |
| Attitude quality improvement (EBPA) | 3.9 | 3.9 – 4.0 | 3.8 | 3.5 – 4.1 | 4.2 | 4.1 – 4.3 | 4.0 | 3.8 – 4.1 | 4.0 | 3.7 - 4.3 | 4.0 | 3.9 – 4.0 |
| Communication/ innovation (TCI) | 3.6 | 3.5 – 3.8 | 3.5 | 2.8 – 4.0 | 3.5 | 3.4 – 3.6 | 3.5 | 3.0 – 3.9 | 4.1 | 4.0 - 4.1 | 3.7 | 3.5 – 3.8 |
| Targets (TCI) | 3.7 | 3.5 – 3.8 | 3.7 | 3.6 – 3.9 | 3.7 | 3.5 – 3.9 | 3.7 | 3.4 – 3.9 | 4.1 | 4.1 | 4.2 | 3.9 – 4.4 |
| Approach- working method (TCI) | 3.5 | 3.2 – 3.6 | 3.6 | 2.9 – 4.2 | 3.3 | 3.2 – 3.3 | 3.3 | 3.0 – 3.5 | 3.7 | 3.8 – 3.6 | 3.8 | 3.5 – 4.0 |
| Attitude guidelines, factor innovation | 7.9 | 0 – 8.0 | 6.7 | 6.0 – 7.0 | 7.8 | 7.5 – 8.0 | 7.6 | 7.0 – 8.0 | 8.0 | 7.4 – 8.5 | 7.9 | 7.7 – 8.0 |
| *Organizational context (average item score):* | | | | | | | | | | | | |
| Organizational conditions present | 4.3 | 3.8 – 4.6 | 3.8 | 2.5 – 4.5 | 4.6 | 4.4 – 4.8 | 3.6 | 3.1 – 4.1 | 4.8 | 4.2 - 5.4 | 4.3 | 3.5 – 5.0 |
| Support management | 4.4 | 4.0 – 4.9 | 2.6 | 1.7 – 3.2 | 5.6 | 5.3 – 5.8 | 4.3 | 3.6 – 5.0 | 3.9 | 3.6 – 4.1 | 3.3 | 2.6 – 4.0 |
| -Inspirational leadership (MFLQ) | 3.9 | 3.4 – 4.3 | 3.0 | 2.0 – 3.6 | 4.0 | 3.9 - 4.1 | 3.6 | 3.2 – 4.0 | 4.2 | 3.8 - 4.5 | 3.4 | 0 - 3.4 |
| -Transactional leadership (MFLQ) | 2.6 | 1.9 – 3.2 | 2.4 | 1.4 – 3.1 | 2.8 | 2.6 – 3.0 | 3.0 | 2.8 – 3.1 | 3.0 | 2.9 - 3.1 | 2.5 | 0 – 2.5 |
| -Passive leadership (MFLQ) | 1.8 | 1.0 – 2.2 | 2.3 | 2.0 – 2.7 | 1.8 | 1.7 - 1.8 | 2.0 | 1.7 - 2.2 | 1.6 | 1.4 – 1.8 | 2.5 | 0 - 2.5 |
