# Supplementary material for: Factors associated with the impact of quality improvement collaboratives in mental healthcare: An exploratory study
Source: Implement Sci. 2012 Jan 9;7:1. doi: 10.1186/1748-5908-7-1 (PMC3313876; doi:10.1186/1748-5908-7-1)
Supplement: Additional file 3 — Table 10 Characteristics of most and least successful QI teams in relation to screening/assessment. [file 1748-5908-7-1-S3.DOC]

Table 10 Characteristics of most and least successful QI teams in relation to screening/assessment

| Possible associated factors | Anxiety disorders (N=8) | | | | Dual diagnosis (N=6) | | | |
| --- | --- | --- | --- | --- | --- | --- | --- | --- |
|  | Most successful teams | | Least successful teams | | Most successful teams | | Least successful teams | |
|  | Mean | Range | Mean | Range | Mean | Range | Mean | Range |
| *Team composition* | | | | | | | | |
| Average age | 41.4 | 39.7 – 43 | 45.0 | 44.0 – 46.0 | 37.3 | 34.5 – 40.0 | 41.6 | 41.3 – 41.9 |
| Number of team members | 6.0 | 6.0 | 6.5 | 6.0 – 7.0 | 10.5 | 8.0 – 13.0 | 10.5 | 8.0 – 13.0 |
| Number of different professionals | 4.0 | 4.0 | 4.5 | 4.0 – 5.0 | 4.5 | 4.0 – 5.0 | 5.5 | 5.0 – 6.0 |
| Level of education: |  |  |  |  |  |  |  |  |
| % Master degree | 66.7 | 33.3-100 | 38.1 | 33.3-42.9 | 22.0 | 15.4 – 28.6 | 29.2 | 25.0 – 33.3 |
| % Bachelor degree | 33.4 | 0 – 66.7 | 61.9 | 57.1 – 66.7 | 66.5 | 61.5 – 71 .4 | 48.0 | 33.3 – 62.5 |
| % Associate degree | 0 | 0 | 0 | 0 | 11.6 | 0 – 23.1 | 22.9 | 12.5 – 33.3 |
| Years of practice in this job | 7.7 | 4.7 – 10.6 | 11.9 | 11.5 – 12.1 | 5.7 | 2.5 – 8.8 | 4.9 | 2.1 – 7.7 |
| Years of practice in this organization | 5.3 | 4.7 – 5.8 | 12.8 | 8.6 – 17 | 3.9 | 3.9 | 8.9 | 8 – 9.7 |
| Number of team members with specialized knowledge | 1.0 | 0 – 2.0 | 3.0 | 3.0 | 3.0 | 3.0 | 2.0 | 1.0 – 3.0 |
| Time spent on improvement | 0.1 | 0.09 – 0.14 | 0.14 | 0.02 – 0.70 | 0.11 | 0.07 – 0.14 | 0.08 | 0.13 – 0.23 |
| % Involvement in quality improvement | 47.9 | 45.8 – 50.0 | 43.2 | 40.0 – 46.4 | 33.1 | 28.6 – 37.5 | 67.9 | 50.0 – 85.7 |
| Participation in national program | | | | | | | | |
| % Attendance conferences QI team members | 41.3 | 32.5 – 50.0 | 60.0 | 60.0 | 72.5 | 57.5 – 87.5 | 63.8 | 57.5 – 70.0 |
| % Attendance of conferences by QI team leaders | 87.5 | 75.0 – 100 | 75.0 | 75.0 | 50.0 | 0 – 100 | 37.5 | 0 – 75.0 |
| *Team functioning* | | | | | | | | |
| Social influence (ASE) | 4.0 | 3.8 – 4.2 | 4.0 | 3.9 – 4.0 | 3.8 | 3.4 – 4.1 | 3.9 | 3.6 – 4.1 |
| Efficacy (ASE) | 3.6 | 3.4 – 3.7 | 3.4 | 3.2 – 3.5 | 3.4 | 3.3 – 3.4 | 3.3 | 3.1 – 3.4 |
| Attitude (ASE) | 3.7 | 3.5 – 3.9 | 3.6 | 3.5 – 3.6 | 3.7 | 3.4 – 3.9 | 4.1 | 4.0 – 4.1 |
| Attitude quality improvement (EBPA) | 3.8 | 3.7 – 3.9 | 4.0 | 3.9 – 4.1 | 4.1 | 3.8 – 4.3 | 4.1 | 4.0 – 4.1 |
| Communication/ innovation (TCI) | 3.7 | 3.3 – 4.1 | 3.9 | 3.8 – 4.0 | 3.3 | 3.0 – 3.6 | 3.6 | 3.3 – 3.9 |
| Targets (TCI) | 4.0 | 3.7– 4.2 | 3.8 | 3.6 – 3.9 | 3.7 | 3.4 – 3.9 | 3.9 | 3.9 |
| Approach- working method (TCI) | 3.6 | 3.2 – 3.9 | 4.0 | 3.8 – 4.2 | 3.2 | 3.0– 3.3 | 3.1 | 2.6 – 3.5 |
| Attitude guidelines, factor innovation | 6.8 | 7.3 – 7.5 | 6.5 | 6.0 – 7.0 | 8.0 | 8.0 | 7.4 | 7.0 – 7.8 |
| *Organizational context* | | | | | | | | |
| Organizational conditions present | 4.4 | 4.2 – 4.6 | 4.5 | 4.5 – 4.5 | 4.0 | 3.1 – 4.8 | 4.1 | 4.1 |
| Support management | 4.4 | 4.2 – 4.5 | 3.1 | 3.0 – 3.2 | 4.7 | 3.6 – 5.8 | 4.9 | 4.7 – 5.0 |
| -Inspirational leadership (MFLQ) | 3.6 | 3.2 – 3.9 | 3.6 | 3.5 – 3.6 | 3.6 | 3.2 – 3.9 | 3.7 | 3.4 – 4.0 |
| -Transactional leadership (MFLQ) | 3.2 | 2.8 – 3.5 | 2.9 | 2.7 – 3.1 | 2.7 | 2.8 – 2.6 | 2.9 | 2.7 – 3. |
| -Passive leadership (MFLQ) | 1.8 | 1.5 – 2.0 | 2.1 | 2.0 – 2.1 | 2.0 | 1.7 – 2.2 | 2.4 | 1.7 – 3.1 |
